# FGF-23 Regulates *CYP27B1* Transcription in the Kidney and in Extra-Renal Tissues

**DOI:** 10.1371/journal.pone.0072816

**Published:** 2013-09-03

**Authors:** Ankanee Chanakul, Martin Y. H. Zhang, Andrew Louw, Harvey J. Armbrecht, Walter L. Miller, Anthony A. Portale, Farzana Perwad

**Affiliations:** 1 Department of Pediatrics, University of California San Francisco, San Francisco, California, United States of America; 2 Geriatric Research, Education, and Clinical Center, St. Louis Veterans Affairs Medical Center, St. Louis, Missouri, United States of America; INSERM, France

## Abstract

The mitochondrial enzyme 25-hydroxyvitamin D 1α-hydroxylase, which is encoded by the *CYP27B1* gene, converts 25OHD to the biological active form of vitamin D, 1,25-dihydroxyvitamin D (1,25(OH)_2_D). Renal 1α-hydroxylase activity is the principal determinant of the circulating 1,25(OH)_2_D concentration and enzyme activity is tightly regulated by several factors. Fibroblast growth factor-23 (FGF-23) decreases serum 1,25(OH)_2_D concentrations by suppressing *CYP27B1* mRNA abundance in mice. In extra-renal tissues, 1α-hydroxylase is responsible for local 1,25(OH)_2_D synthesis, which has important paracrine actions, but whether FGF-23 regulates *CYP27B1* gene expression in extra-renal tissues is unknown. We sought to determine whether FGF-23 regulates *CYP27B1* transcription in the kidney and whether extra-renal tissues are target sites for FGF-23-induced suppression of *CYP27B1.* In HEK293 cells transfected with the human *CYP27B1* promoter, FGF-23 suppressed promoter activity by 70%, and the suppressive effect was blocked by CI-1040, a specific inhibitor of extracellular signal regulated kinase 1/2. To examine *CYP27B1* transcriptional activity *in vivo*, we crossed fgf-23 null mice with mice bearing the *CYP27B1* promoter-driven luciferase transgene (1α-Luc). In the kidney of FGF-23 null/1α-Luc mice, *CYP27B1* promoter activity was increased by 3-fold compared to that in wild-type/1α-Luc mice. Intraperitoneal injection of FGF-23 suppressed renal *CYP27B1* promoter activity and protein expression by 26% and 60% respectively, and the suppressive effect was blocked by PD0325901, an ERK1/2 inhibitor. These findings provide evidence that FGF-23 suppresses *CYP27B1* transcription in the kidney. Furthermore, we demonstrate that in FGF-23 null/1α-Luc mice, *CYP27B1* promoter activity and mRNA abundance are increased in several extra-renal sites. In the heart of FGF-23 null/1α-Luc mice, *CYP27B1* promoter activity and mRNA were 2- and 5-fold higher, respectively, than in control mice. We also observed a 3- to 10-fold increase in *CYP27B1* mRNA abundance in the lung, spleen, aorta and testis of FGF-23 null/1α-Luc mice. Thus, we have identified novel extra-renal target sites for FGF-23-mediated regulation of *CYP27B1*.

## Introduction

Vitamin D is activated by sequential hydroxylations in the liver and kidney to produce the active form of vitamin D; 1,25 dihydroxyvitamin D (1,25(OH)_2_D), which plays a central role in calcium and phosphorus homeostasis, skeletal growth and mineralization, and tissue differentiation [1]. In the liver, the 25-hydroxylation of vitamin D is catalyzed by several enzymes including the mitochondrial enzyme CYP27A1 [2] and the microsomal enzyme CYP2R1 [3]. Hepatic synthesis of 25 hydroxyvitamin D (25OHD) is dependent on substrate concentration, which is determined by dietary intake of vitamin D and exposure to sunlight. In the kidney, 1,25(OH)_2_D is produced by the mitochondrial enzyme, 25-hydroxyvitamin D 1α-hydroxylase (1α-hydroxylase) [4]–[9]. 1α-hydroxylation is the rate-limiting step in the bioactivation of vitamin D. Human, rat and mouse 1α-hydroxylase (*CYP27B1*) genes have been cloned [10]–[15], and inactivating mutations in *CYP27B1* have been identified in patients with autosomal recessive 1α-hydroxylase deficiency, also known as vitamin D dependent rickets type I A [10], [11], [16]–[18]. Vitamin D 24-hydroxylase (24-hydroxylase) catalyzes the 24-hydroxylation of 25OHD to 24,25(OH)_2_D and of 1,25(OH)_2_D to 1,24,25(OH)_3_D; both reactions initiate the metabolic inactivation of vitamin D via the C24-oxidation pathway [19], [20]. 24-hydroxylase activity is found in kidney, intestine, skin, macrophages and other tissues [21]. Circulating concentrations of 1,25(OH)_2_D primarily reflect its synthesis in the kidney; however, 1α-hydroxylase activity is also found in keratinocytes, macrophages, osteoblasts and other tissues [22]–[24]. Bilateral nephrectomy and chronic kidney disease result in low circulating 1,25(OH)_2_D concentrations, suggesting that extra-renal synthesis of 1,25(OH)_2_D contributes little to the maintenance of normal serum 1,25(OH)_2_D concentrations [25]. However, 1α-hydroxylase activity in extra-renal tissues is an important source of 1,25(OH)_2_D for its autocrine and paracrine functions in bone, skin, macrophages, prostate, parathyroid and several other tissues [26]–[30].

In the renal proximal tubule, 1α-hydroxylase activity is tightly regulated by parathyroid hormone (PTH), calcium (Ca), phosphorus (Pi) and 1,25(OH)_2_D [31], [32]. Recently, we and others demonstrated that fibroblast growth factor-23 (FGF-23), a bone-derived circulating peptide, is a critical determinant of the renal metabolism of vitamin D [33]–[37]. FGF-23 suppresses renal 1,25(OH)_2_D production by suppressing *CYP27B1* and stimulating 24-hydroxylase *(CYP24A1)* mRNA expression in the kidney in normal mice [35], [37]. We showed in cultured human and mouse renal proximal tubule epithelia that FGF-23 can directly suppress *CYP27B1* expression and that this effect is mediated by activation of the mitogen activated protein kinase signaling pathway (MAPK) [35]. However, whether FGF-23 regulates *CYP27B1* expression by transcriptional or post-transcriptional mechanisms is unknown.

The human *CYP27B1* gene is located on chromosome 12q13.1-q13.3 and consists of 9 exons spanning approximately 6.5 kb in length [11]. Regulatory elements within 1.5 kb of the 5′ flanking region of *CYP27B1* are important for regulation by PTH, calcitonin, and 1,25(OH)_2_D [38]–[40] in renal tissue. Whether FGF-23 regulates *CYP27B1* in extra-renal sites is unknown. To understand further the molecular mechanisms by which FGF-23 regulates *CYP27B1* and the sites involved, we examined transcriptional regulation of *CYP27B1* by FGF-23 both *in vitro* and *in vivo*. In the present study, we show that *CYP27B1* transcription is regulated by FGF-23 in the kidney and in extra-renal sites and thus identify novel tissue targets for FGF-23 action.

## Materials and Methods

### Cell Culture and Transfection

Human embryonic kidney (HEK-293) cells stably transfected with the transmembrane (Tm) form of mouse klotho-pEF1 expression vector [41] (a kind gift from Makoto Kuro-o, University of Texas Southwestern, Dallas, TX), were grown and maintained as previously described [41]. Klotho is an obligatory co-factor for FGF-23 and confers tissue specificity for it actions in target tissues [42]. It is well established that FGF-23-dependent signal activation in HEK-293 cells is dependent on Tm klotho [41], [42]. Based on this prior work, we chose to perform all our experiments in HEK293 cells stably transfected with Tm klotho. HEK-293 cells were plated at 130,000/well in 24-well plates, in DMEM H-21 with 10% FBS (Hyclone, Waltham, MA). At 80% confluence, cells were transiently transfected with 1 µg DNA/well of pGL-3 basic vector or *CYP27B1* promoter-driven firefly luciferase reporter plasmid using 2 µl/well of Lipofectamine 2000 (Invitrogen, Carlsbad, CA) as per manufacturer’s protocol. Cells were co-transfected with 50 ng/well of pRL-CMV Vector (Promega, Madison, WI) containing the *Renilla* luciferase gene driven by the CMV promoter to normalize for transfection efficiency. HEK-293 cells were then treated with varying doses of FGF-23 for 21 hours in serum-free media. For the inhibitor experiments, CI-1040 (Pfizer, New York, NY), a selective MAPK kinase (MEK) inhibitor, that blocks phosphorylation of extracellular signal regulated kinase 1/2 (ERK1/2) was added 1 hour before treatment with FGF-23. Mouse aortic vascular smooth muscle cells (VSMC) were purchased from ATCC, VA, cultured in 6-well plates in DMEM H-21 with 10% FBS. At 80% confluence, cells were treated with FGF-23 for 5–30 minutes to determine activation of MAPK signaling pathway, and for 21 hours, to determine *CYP27B1* mRNA expression.

### Plasmid Constructs

The cloning and sequencing of the human *CYP27B1* gene, and the localization of its transcriptional start site 62 bp upstream from ATG translational start site have been described [11]. A 5′ fragment extending from the *Eco*RI site at –345 bp to the translational start site was prepared by PCR and cloned into pGL-3-basic vector (Promega) containing the luciferase reporter gene, in-frame with the ATG site to obtain the 409 bp *CYP27B1* promoter plasmid. To obtain longer fragments of 5′ flanking DNA, human genomic DNA was digested with *Bam*HI, cloned into pcDNA 3.1 (Invitrogen Carlsbad, CA) and screened with the 5′ *Eco*RI/*Nhe*I to identify a clone with additional 5′ flanking DNA. The *Bam*HI/*Eco*RI fragment of this clone extending from –1151 bp to –345 bp was inserted upstream from the –345 bp in the pGL-3-basic vector to obtain the “full-length” 1576 bp *CYP27B1* promoter plasmid. Subsequent digestions with *Nhe*I, *Sma*I, *Hind*III, *Tth*III1 and *Xho1* yielded additional deletion constructs containing −1171, −926, –789 and −200 bp, respectively. Egr-1 promoter driven luciferase plasmid was a kind gift from Kirin Pharma, Japan. pRL-CMV plasmid was purchased from Promega.

### FGF-23

Recombinant human FGF-23(R176Q) (Genzyme Corporation, Framingham, MA) contains a mutation in its proprotein convertase (furin) proteolytic cleavage site in which arginine at position 176 is replaced by glutamine. This mutation is identical to that found in patients with autosomal dominant hypophosphatemic rickets and renders the FGF-23(R176Q) protein resistant to proteolytic processing [43]. FGF-23(R176Q) has enhanced biological potency *in vivo* and *in vitro* when compared with that of native FGF-23 [44]. Hereforth, recombinant human FGF-23(R176Q) will be referred to as FGF-23.

### Animals

pCYP27B1(–1501 bp)-Luc transgenic mice were generated and bred as described previously [45]. The pGL3-pCYP27B1-luciferase (pGL3-pCYP27B1-luc) plasmid construct consists of the luciferase reporter gene flanked by the 1501 bp promoter region of the human 1α-hydroxylase gene. Briefly, the transgenic mice were generated by pro-nuclear injection of the purified pGL3-pCYP27B1-luc construct into CBA/C57 embryos to generate founder mice and further backcrossed more than 10 generations into a C57/Bl6 background. Hereforth, the pCYP27B1 (–1501 bp)-Luc transgenic mice will be referred to as 1α-Luc transgenic mice. The 1α-Luc transgenic mouse model is ideal to study the effect of various physiological stimuli; these mice demonstrate an appropriate increase in *CYP27B1* promoter-driven luciferase activity in response to dietary restriction of calcium and vitamin D. In addition, promoter activity in these mice closely correlates with *CYP27B1* mRNA and protein expression in renal and extra-renal tissues [46], [47].

*Fgf-23* null mice (*fgf-23^−/−^*) were generated and bred as previously described [48], [49]. At an early age, *fgf-23^−/−^* mice develop hyperphosphatemia and increased serum 1,25 (OH)_2_D concentrations, the latter due to greatly increased renal expression of *CYP27B1* mRNA and thereby increased renal 1,25(OH)_2_D synthesis. To generate the double mutant mouse, we crossbred mice heterozygous for fgf-23 gene (*fgf-23^+/−^*) and 1α-Luc gene (1α-Luc*^+/−^*) to obtain *fgf-23^+/−/^*1α-Luc*^+/−^* transgenic mice. *Fgf-23^+/−/^*1α-Luc*^+/−^* mice were mated with *fgf-23^+/−/^*1α-Luc*^−/−^* to obtain *fgf-23^−/−/^*1α-Luc*^+/−^* transgenic mice. *Fgf-23^+/+^/*1α-Luc*^+/−^*, *fgf-23^+/−/^*1α-Luc*^+/−^* and *fgf-23^−/−/^*1α-Luc*^+/−^* transgenic were studied at 28-35 days of age. To determine the effect of FGF-23 and MAPK inhibitor treatment on *CYP27B1* expression *in vivo, fgf-23^−/−/^*1α-Luc*^+/−^* mice were fed a constant diet containing 0.6% phosphorus and 0.6% calcium (Teklad diet 98243, Harlan Laboratories, Madison, WI) and received the MEK inhibitor, PD0325901, 12.5 mg/kg/dose, or vehicle orally for 4 days. On day 5, a single intraperitoneal injection of FGF-23 (150 ng/g) was administered and mice were sacrificed 5 hours later and tissues were removed and frozen for subsequent preparation of total RNA and protein. To determine whether regulation of *CYP27B1* expression was specific to FGF-23 action, fgf*-23^+/+^/*1α-Luc^+*/−*^ mice were administered a single intraperitoneal injection of basic FGF (250 ng/g) and mice were sacrificed 5 hours later. All procedures were approved by the Committee on Animal Research, University of California San Francisco.

### Real-Time PCR

Total RNA was isolated from tissues using TRIzol reagent (Invitrogen). cDNA was synthesized using 1×PCR buffer, 7.5 mM MgCl_2_, 1 mM dNTP, 5 µM random primers, and 2.5 U/L Moloney murine leukemia virus reverse transcriptase (Invitrogen) at the following temperatures: 25°C for 10 min, 48°C for 40 min, and 95°C for 5 min. The probes and primers for mouse *CYP27B1*, *CYP24A1* and β-glucuronidase (Gus) were custom designed as previously described [50]. The mRNA abundance of the gene of interest, expressed relative to that of Gus mRNA, was quantitated by real-time PCR using the ABI 7900 HT Sequence Detection System (Applied Biosystems, Foster City, CA) as previously described [35]. The threshold cycle (Ct) at which a statistically significant increase in signal above background fluorescence was determined, and the Ct values for the gene of interest were normalized to Ct values for Gus.

### Western Blot Analysis

Mouse kidney total protein (25 µg), and renal mitochondrial protein (35 µg) were isolated, fractionated on 10% SDS-polyacrylamide gel and transferred to polyvinilidine difluoride membranes (PVDF) membranes (BioRad, Hercules, CA) as previously described [50]. Activation of MAP kinase signaling in the kidney was determined by detection of pERK1/2 protein using anti-phospho-ERK1/2 monoclonal antibody (Cell Signaling Technology, Danvers, MA) and visualized by chemiluminescence (Supersignal West Dura, Pierce Biotechnology, Rockford, IL). Equal protein loading was determined using a mouse anti-ERK 2 monoclonal antibody (Santa Cruz Biotechnology, Inc., Santa Cruz, CA). For detection of 1α-hydroxylase protein, membranes were probed with rabbit anti-1α-hydroxylase polyclonal antibody (1∶1500 dilution) [51]. Equal protein loading was determined using a rabbit anti-β-actin polyclonal antibody (1∶6500) (Cell Signaling Technology, Danvers, MA). The membranes were subsequently blotted using an infrared (IR) labeled secondary antibody, IR Dye 800 CW and IR Dye 680 (1∶25,000) (Li-Cor Biosciences, Lincoln, NE). The bound complex was detected using Odyssey Infrared Imaging System (LiCor Biotechnology). The images were analyzed using the Odyssey Application Software to obtain the integrated fluorescence intensities from IR detection. The ratio of fluorescence intensities of the protein of interest was normalized to that of the loading control protein and the data were plotted on a bar graph.

### Luciferase Activity

HEK 293 cells were lysed using passive lysis buffer from the dual luciferase assay kit (Promega, Madison, WI). 10 µl of lysate was used to quantify firefly luciferase activity using a TD 20/20 luminometer (Turner Design Instruments, Sunnyvale, CA). Luciferase activity was normalized by calculating the ratio of firefly luciferase to renilla luciferase activity per sample and results expressed as fold change of FGF-23-treated to vehicle-treated samples. Experiments were performed in triplicate and repeated 3 times. For mouse samples, tissues were homogenized in passive lysis buffer and centrifuged at 15,000 g for 5 min at 4°C as previously described [47]. Firefly luciferase activity was measured in the supernatant in duplicate using the Luciferase Assay System (Promega). A volume of 100 µl of supernatant was combined with 50 µl of Luciferase Assay Reagent and the luminescence signal was measured using the Victor luminometer (PERKinElmer, Waltham, MA). All luciferase measurements were corrected for auto-luminescence by subtracting the luminescence signal measured in a mixture of 100 µl of passive lysis buffer and 50 µl of Luciferase Assay Reagent. The total protein content of the tissue supernatants was measured using the BCA Protein Assay Kit (Bio-Rad). The activity of the 1α-hydroxylase promoter was expressed as the enzymatic luciferase activity per µg of total cellular protein.

### Serum Biochemistry

Serum Ca and Pi concentrations were determined using kits from Stanbio Laboratories (San Antonio, TX). Serum intact PTH concentrations were determined using EIA kits from Immutopics International (San Clemente, CA).

### Statistical Analysis

Data are expressed as mean ± SEM. The significance of differences between two groups was analyzed by student t test or between multiple groups by analysis of variance (ANOVA). Post-hoc analyses were performed using the Bonferroni method. *P* value <0.05 was considered as statistically significant.

## Results

### Basal Activity of the Human *CYP27B1* Promoter

**Figure 1:**
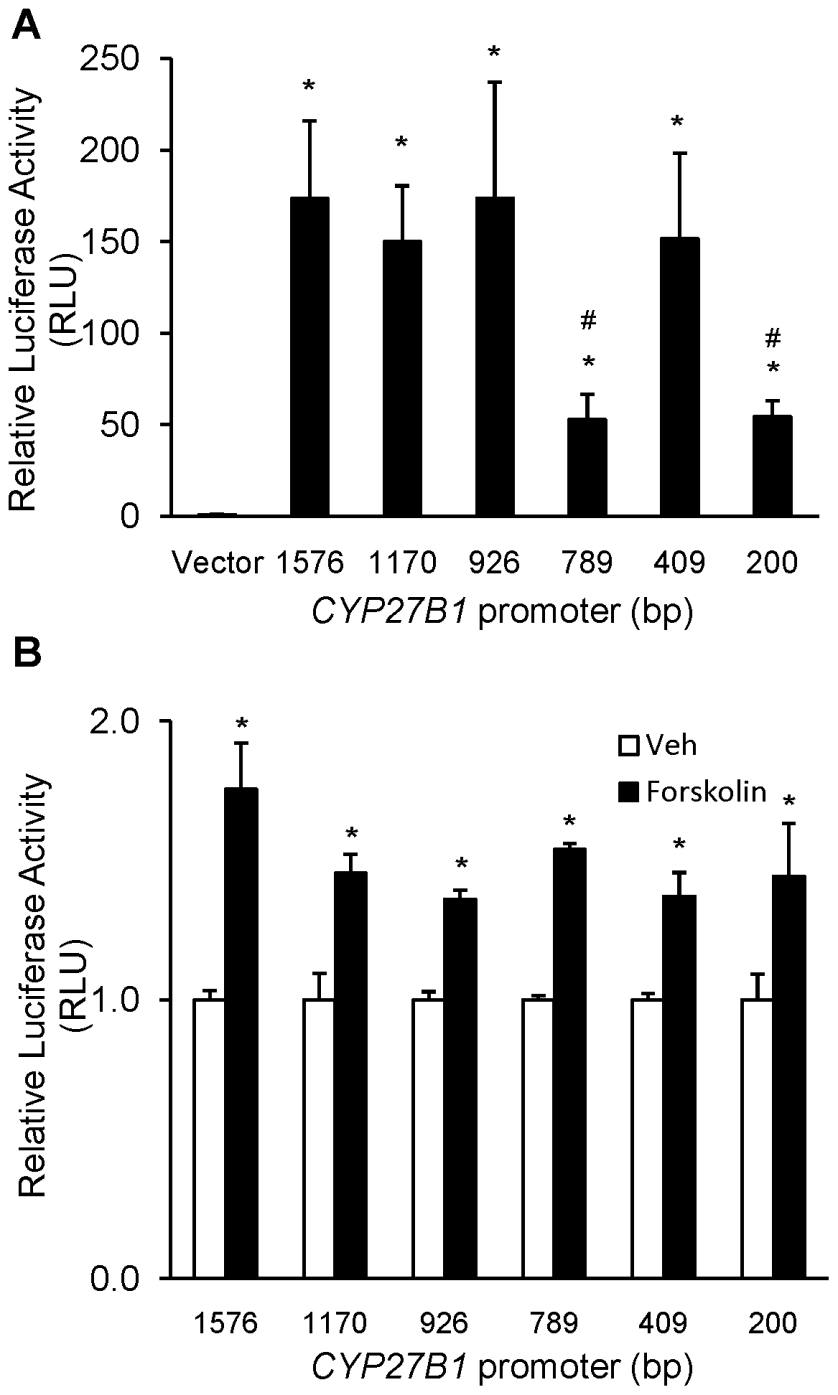
A. Basal activity of the human *CYP27B1* promoter constructs (−1576 bp, −1170 bp, −926 bp, −789 bp, −409 bp, −200 bp) in HEK293 cells. 1α-hydroxylase promoter-driven firefly luciferase activity is normalized to renilla luciferase activity in each individual sample and expressed as relative luciferase activity units (RLU). *P<0.05 when compared to HEK293 cells transfected with empty vector. ^#^ P<0.05 when compared to HEK293 cells transfected with 1.6 kb *CYP27B1* promoter construct. B. Effect of forskolin (10^−5^ M) on *CYP27B1* promoter activity in HEK293 cells. Bars depict fold induction of relative luciferase activity expressed as mean±SEM. *P<0.05 when compared to HEK293 cells treated with vehicle for each promoter deletion construct. (n = 3 experiments, each performed in triplicate).

We generated a 1576 bp *CYP27B1* promoter fragment from PCR reactions using appropriate primers and human genomic DNA as template and we tested the functional activity of the *CYP27B1* promoter in transiently transfected HEK-293 cells. Luciferase activity of the full-length (−1576 bp) promoter was substantially (170-fold) higher than that of the promoterless vector, and increased luciferase activity was sustained in the −1.1 kb and −926 bp deletion constructs (*P*<0.05) (Fig. 1A). Promoter activity decreased by 70% in cells transfected with the 789bp deletion construct as compared to the full-length promoter, suggesting the presence of an enhancer between −926 bp and −789 bp. Promoter activity increased with further shortening of the promoter to −409 bp suggesting the presence of a silencer between −789 and −409 bp. With the −200 bp construct, promoter activity was 50-fold higher than the vector control but considerably reduced compared to the full-length promoter activity (*P*<0.05). We then determined the response of the *CYP27B1* promoter to stimulation by forskolin which increases intracellular cAMP and stimulates 1α-hydroxylase promoter activity in opossum kidney cell cultures [52]. With forskolin treatment, activity of the full-length CYP27B1 promoter increased by 76% compared to the vehicle-treated cells. The stimulatory effect of forskolin was sustained in all the deletion constructs (Fig. 1B).

### Effect of FGF-23 on *CYP27B1* Promoter Activity *in vitro*

**Figure 2:**
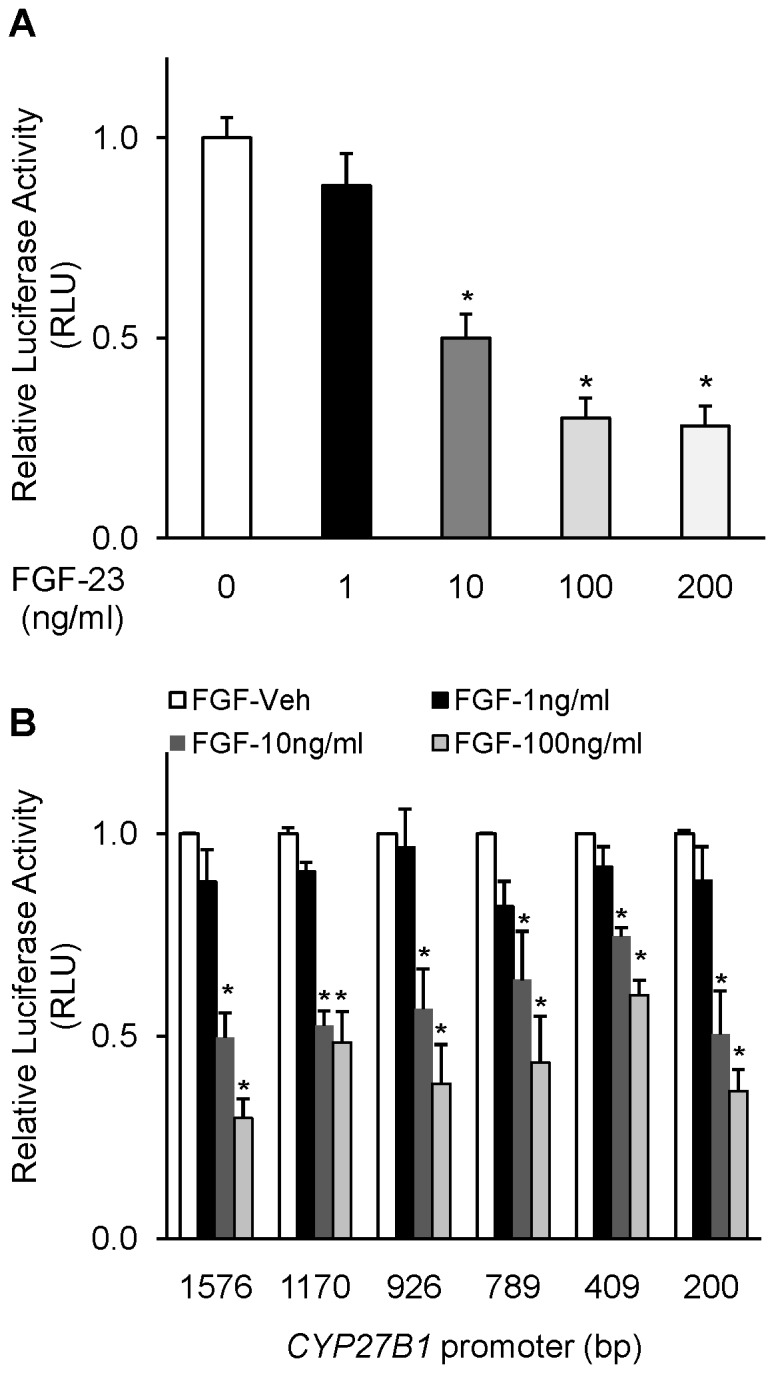
Effect of FGF-23 on CYP27B1 promoter activity in HEK293 cells. HEK293 cells were transfected with CYP27B1 promoter constructs and treated with FGF-23 (1–200 ng/ml) or vehicle for 21 hours. **A.** 1576 bp full-length CYP27B1 promoter-driven luciferase activity normalized to renilla luciferase activity and expressed as fold change to vehicle-treated samples. **B.** CYP27B1 promoter activity of the deletion constructs (−1576 bp, −1170 bp, −926 bp, −789 bp, −409 bp, −200 bp), normalized to renilla luciferase activity. Bars depict fold change of luciferase activity relative to vehicle-treated samples, expressed as mean±SEM (n = 3 experiments, each performed in triplicate). *P<0.05 when compared to the vehicle group.

To determine whether FGF-23 transcriptionally regulates the *CYP27B1* gene, we used HEK-293 cells stably transfected with klotho and expressing a *CYP27B1* promoter/luciferase-reporter construct. Treatment of cells with recombinant human FGF-23 elicited a dose-dependent suppression of luciferase activity that was maximal (70%) at a dose of 100 ng/ml (Fig. 2A). Higher concentrations of FGF-23 did not suppress promoter activity further. To identify the responsible regulatory region of the *CYP27B1* promoter, we treated HEK 293 cells transfected with *CYP27B1* promoter-deletion constructs with FGF-23 (100 ng/ml). FGF-23 suppressed *CYP27B1* promoter activity in all the deletion constructs, although the fold-change of suppression varied with each construct (Fig. 2B). These findings provide evidence that FGF23 regulates *CYP27B1* at the transcriptional level and suggests that the FGF-23-responsive regulatory region lies within 200 bp of the *CYP27B1* transcription start site. To determine whether klotho is required for the regulation of *CYP27B1* expression by FGF-23 in HEK-293 cells, we determined *CYP27B1* mRNA expression and *CYP27B1* promoter activity in HEK-293 cells that were not stably transfected with klotho. In the absence of klotho, treatment of HEK-293 cells with FGF-23 did not elicit a significant suppression of *CYP27B1* mRNA expression (100.4±2.6 (vehicle) vs 108.0±7.0 (FGF-23 (10 ng/ml), P = 0.18) or promoter activity (104.2±23.4 (vehicle) vs 74.8±10.6 (FGF-23 (10 ng/ml), P = 0.17).

### Role of MAP Kinase Signaling Pathway in the Transcriptional Regulation of *CYP27B1* by FGF-23 *in vitro*

**Figure 3:**
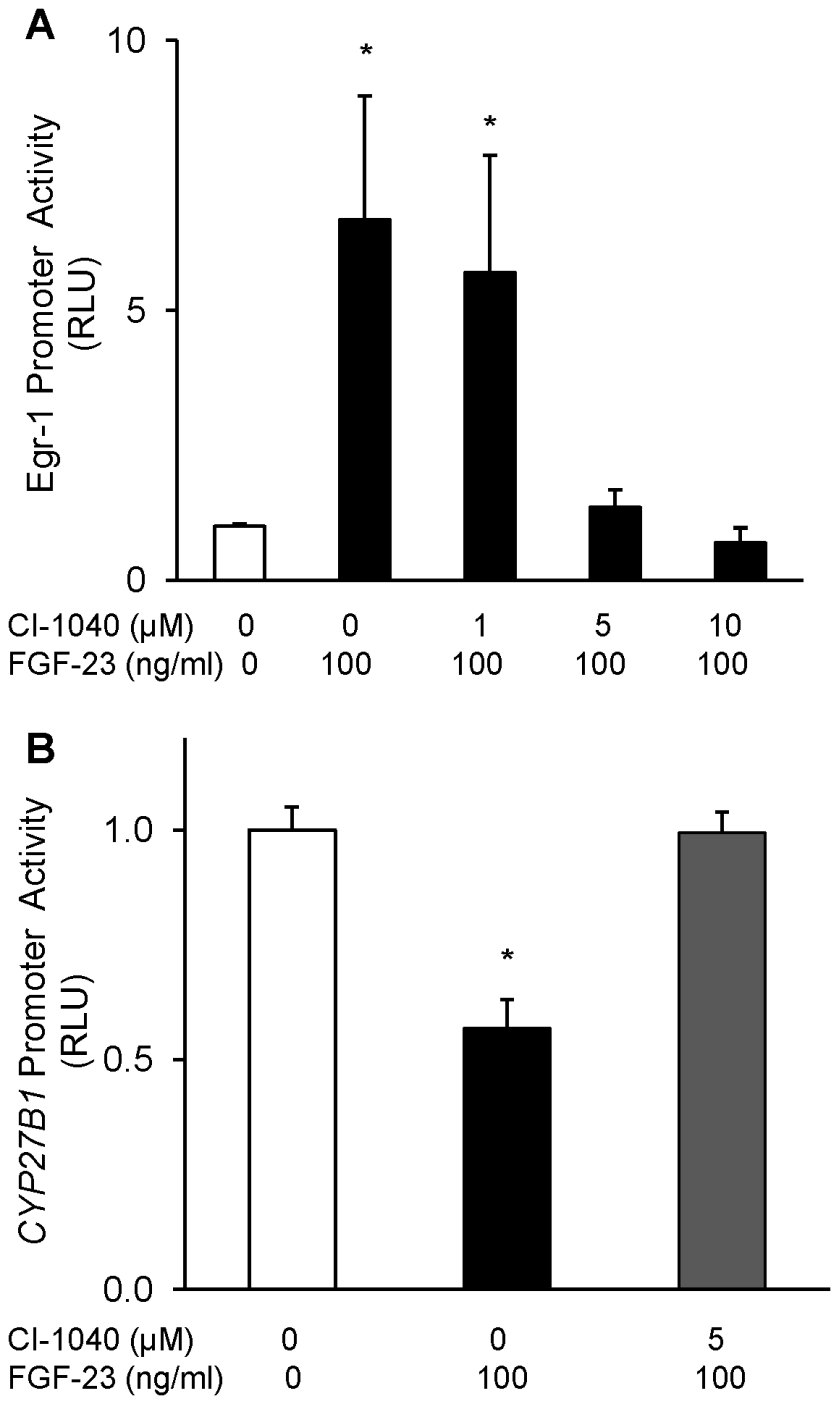
Role of MEK/ERK1/2 pathway in FGF-23-mediated signaling in HEK293 cells. HEK293 cells were transfected with Egr-1 promoter or −200 bp CYP27B1 promoter plasmid and treated with FGF-23 (100 ng/ml) or vehicle for 21 hours. For MEK inhibition, HEK-293 cells were pre-treated with CI-1040 (0–10 µM) 30 min prior to FGF-23 treatment. **A.** Egr-1 promoter-driven luciferase activity normalized to renilla luciferase activity and expressed as fold change to vehicle-treated samples. **B.** CYP27B1 promoter activity of the −200 bp deletion construct normalized to renilla luciferase activity and expressed as fold change to vehicle-treated samples. Bars depict mean±SEM (n = 3 experiments, each performed in triplicate). *P<0.05 when compared to the vehicle group.

We previously demonstrated that the suppression of *CYP27B1* mRNA expression by FGF-23 depends upon MAPK signaling via ERK1/2 [35], [50], [53]. Acute activation of this pathway is evidenced by phosphorylation of ERK1/2 and increased expression of the transcription factor, early growth response-1 (egr-1). When we treated HEK-293 cells transfected with egr-1 promoter-driven luciferase plasmid with FGF-23 (100 ng/ml), egr-1 promoter activity was stimulated by 7-fold compared to vehicle-treated cells. Pre-treatment with a specific MEK inhibitor, CI-1040, blocked the stimulation of egr-1 promoter activity by FGF-23 at higher doses (5 and 10 µM) but showed no effect at lowest dose (1 µM) (Fig. 3A). These findings confirm the activation of ERK1/2 signaling in HEK-293 cells by FGF-23. To determine whether transcriptional regulation of *CYP27B1* by FGF-23 depends upon activation of MAPK signaling, we treated HEK-293 cells transfected with the −200 bp *CYP27B1* promoter construct with FGF-23, with and without prior treatment with CI-1040. FGF-23 suppressed *CYP27B1* promoter activity by 43%, but the suppressive effect was completely blocked by pre-treatment with CI-1040 (Fig. 3B).

### Role of FGF-23 in the Transcriptional Regulation of *CYP27B1* Gene in Mice

**Figure 4:**
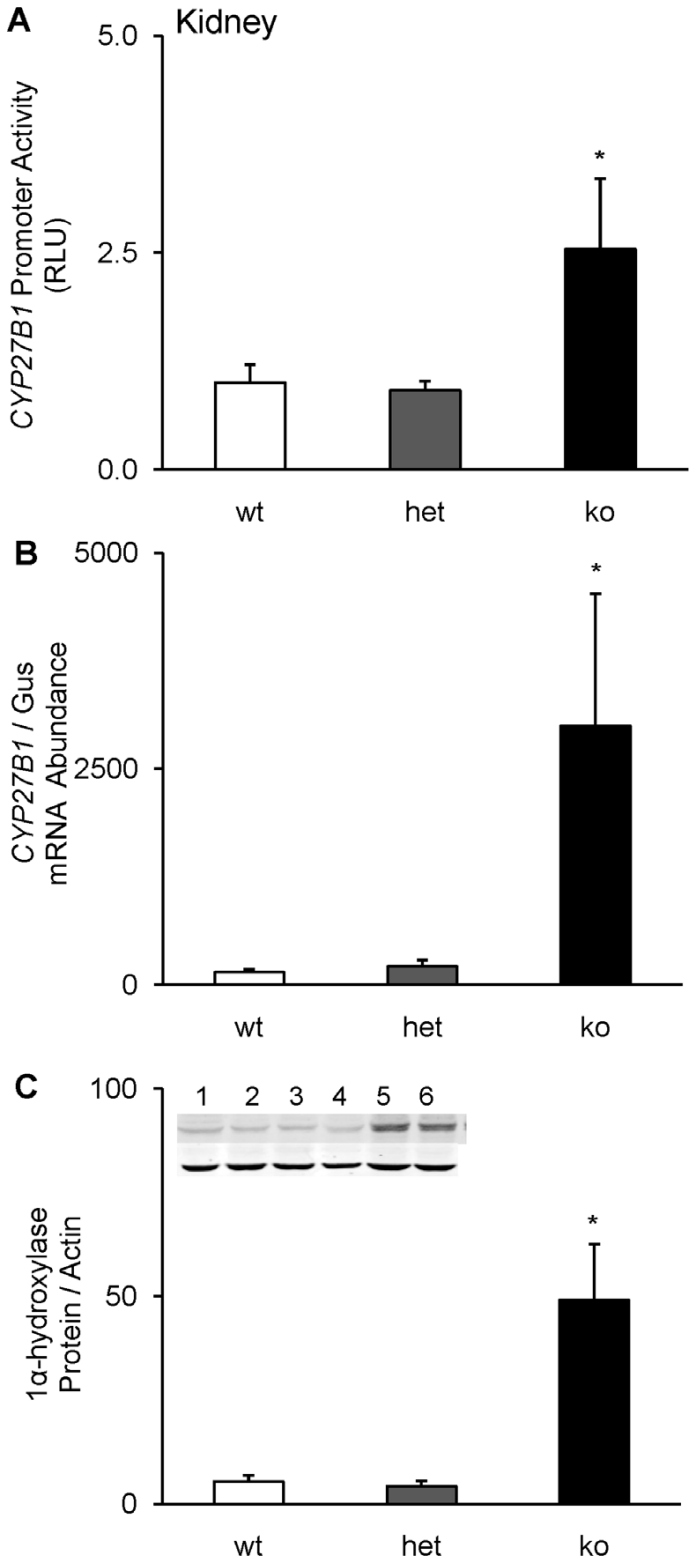
Renal expression of *CYP27B1*. *Fgf-23^+/+^/*1α-Luc*^+/−^* (wt), fgf*-23^+/−/^*1α-Luc*^+/−^* (het) and *fgf-23^−/−/^*1α-Luc*^+/−^* (ko) mice were bred as described in *Methods.* Mice were sacrificed at 4 weeks of age and the kidneys removed and divided into three parts for determination of **A.** *CYP27B1* promoter activity, expressed as luciferase activity per mg of tissue. Graph depicts fold change with respect to luciferase activity in *fgf-23^+/+^/*1α-Luc*^+/−^* mice. **B.** *CYP27B1* mRNA expression, quantitated by real-time PCR, normalized to that of gus mRNA, and expressed as a percent relative to *fgf-23^+/+^/*1α-Luc*^+/−^* mice. **C.** Renal mitochondrial 1α-hydroxylase protein abundance normalized to β-actin (for western blotting, n = 1 mouse/lane). Lane 1–2 (*fgf-23^+/+^/*1α-Luc*^+/−^* mice), 3–4 (*fgf-23^+/−/^*1α-Luc*^+/−^* mice), 5–6 (*fgf-23^−/−/^*1α-Luc*^+/−^* mice). Bars depict mean±SEM (n = 5 mice/group), *P<0.05, compared to *fgf-23^+/+^/*1α-Luc*^+/−^* mice.

Targeted ablation of the *fgf-23 *gene in mice disrupts calcium, phosphorus and 1,25 (OH)_2_D homeostasis [34], [48], giving rise to hypercalcemia, hyperphosphatemia and increased serum 1,25(OH)_2_D concentrations, the latter due to significantly increased renal expression of *CYP27B1* mRNA and protein. To determine whether the increased expression of *CYP27B1* mRNA and protein in fgf23 null mice is due to an increase in gene transcription, we measured *CYP27B1* promoter-driven luciferase activity, mRNA and protein expression in the kidney in *fgf-23^+/+^/*1α-Luc*^+/−^*, *fgf-23^+/−/^*1α-Luc*^+/−^* and *fgf-23^−/−/^*1α-Luc*^+/−^* transgenic mice at 3 weeks of age. Luciferase activity in the kidney was 2.5-fold higher in *fgf-23^−/−/^*1α-Luc*^+/−^* mice than in control mice (*fgf-23^+/+^/*1α-Luc*^+/−^*) (Fig. 4A). We detected no significant change in promoter activity in the heterozygous (*fgf-23^+/−/^*1α-Luc*^+/−^*) mice compared to the control group. Renal expression of *CYP27B1* mRNA and protein were upregulated by 300- and 50-fold, respectively, in *fgf-23^−/−/^*1α-Luc*^+/−^* transgenic mice (Fig. 4B and C). These findings provide evidence that up-regulation of *CYP27B1* in the kidney in fgf-23 null mice is mediated at least in part by an increase in gene transcription.

### Role of MAP Kinase Signaling in the Transcriptional Regulation of *CYP27B1* by FGF-23 in Mice

**Figure 5:**
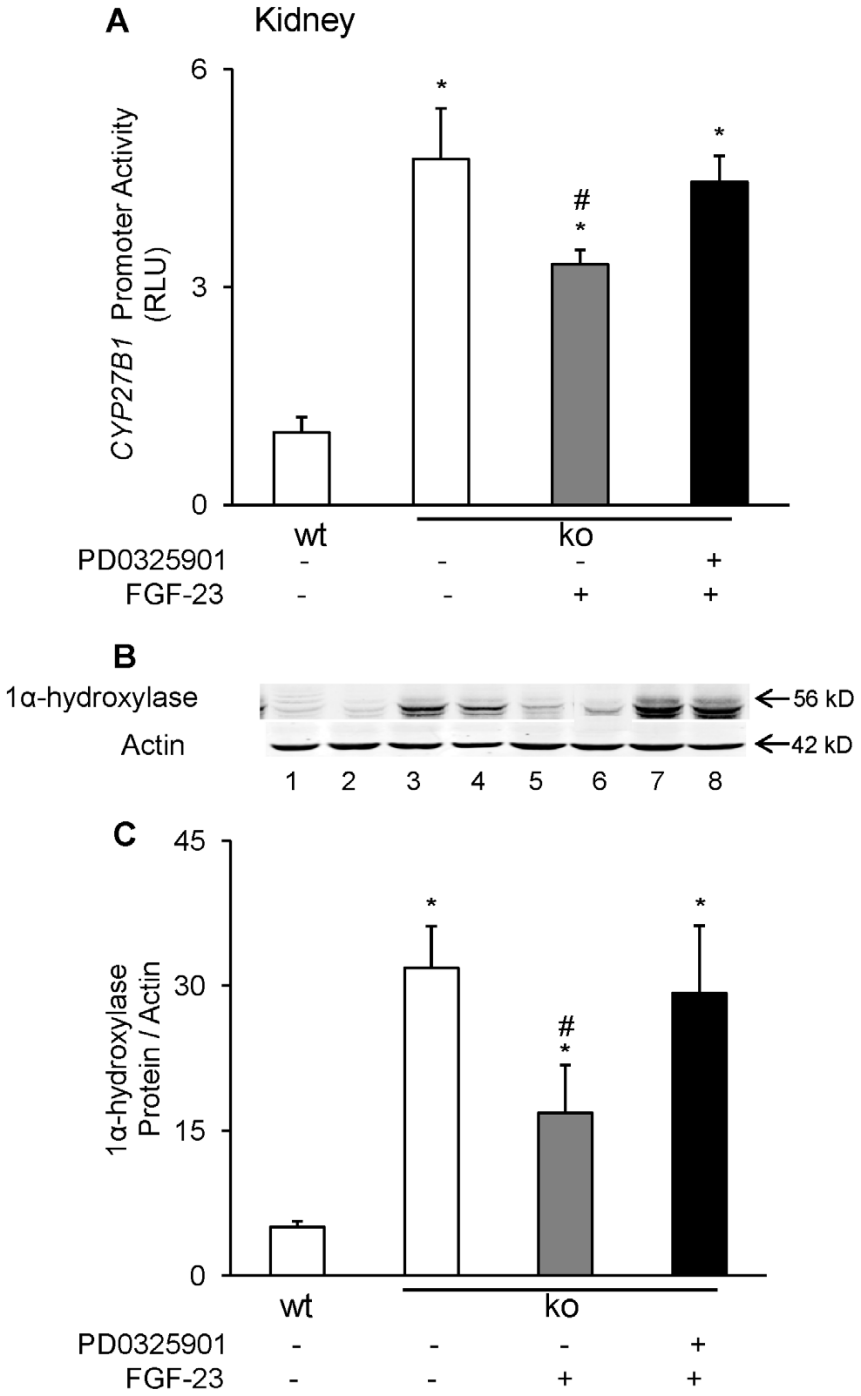
Effects of MEK/ERK1/2 signaling blockade on *CYP27B1* in the kidney. *Fgf-23^+/+^/*1α-Luc*^+/−^* (wt) and *fgf-23^−/−/^*1α-Luc*^+/−^* (ko) transgenic mice were treated with vehicle or PD0325901 and administered a single injection of FGF-23 as described in *Methods*. **A.** Renal *CYP27B1* activity, expressed as luciferase activity per mg of tissue. Graph depicts fold change with respect to luciferase activity in vehicle-treated *fgf-23^+/+^/*1α-Luc mice. **B.** Renal mitochondrial 1α-hydroxylase protein abundance normalized to β-actin (for western blotting, n = 1 mouse/lane). Lane 1–2 (vehicle-treated *fgf-23^+/+^/*1α-Luc*^+/−^* mice), 3–4 (vehicle-treated *fgf-23^−/−/^*1α-Luc*^+/−^* mice), 5–6 (FGF-23-treated *fgf-23^−/−/^*1α-Luc*^+/−^* mice), 7–8 (FGF-23+PD0325901-treated *fgf-23^−/−/^*1α-Luc*^+/−^* mice). Bars depict mean±SEM (n = 5 mice/group. *P<0.05, compared to vehicle-treated *fgf-23^+/+^/*1α-Luc*^+/−^* mice; ^#^ *P<0.05*, compared to vehicle-treated *fgf-23^−/−/^*1α-Luc*^+/−^* mice.

In a mouse model of FGF-23 excess, we showed that FGF-23-induced suppression of renal *CYP27B1* mRNA and protein was critically dependent upon MAPK signaling [50], [53]. To determine *in vivo* whether suppression of *CYP27B1* gene transcription by FGF-23 depends upon MAPK signaling, we administered FGF-23 to *fgf-23^−/−/^*1α-Luc^+*/−*^ transgenic mice that were pre-treated with the MEK inhibitor, PD0325901. FGF-23 treatment suppressed *CYP27B1* promoter-driven luciferase activity in *fgf-23^−/−/^*1α-Luc^+*/−*^ transgenic mice by 26%, and this suppressive effect was blocked by pre-treatment with PD0325901 (Fig. 5A). Simultaneously, FGF-23 treatment suppressed renal mitochondrial 1α-hydroxylase protein by 60%, and this effect was also blocked by PD0325901 (Fig. 5B). These studies provide evidence that the suppression of *CYP27B1* transcription by FGF-23 *in vivo* is dependent on activation of MAPK signaling via ERK1/2.

### Role of FGF-23 in the Regulation of *CYP27B1* in Extra-renal Sites

#### Cardiovascular

**Figure 6:**
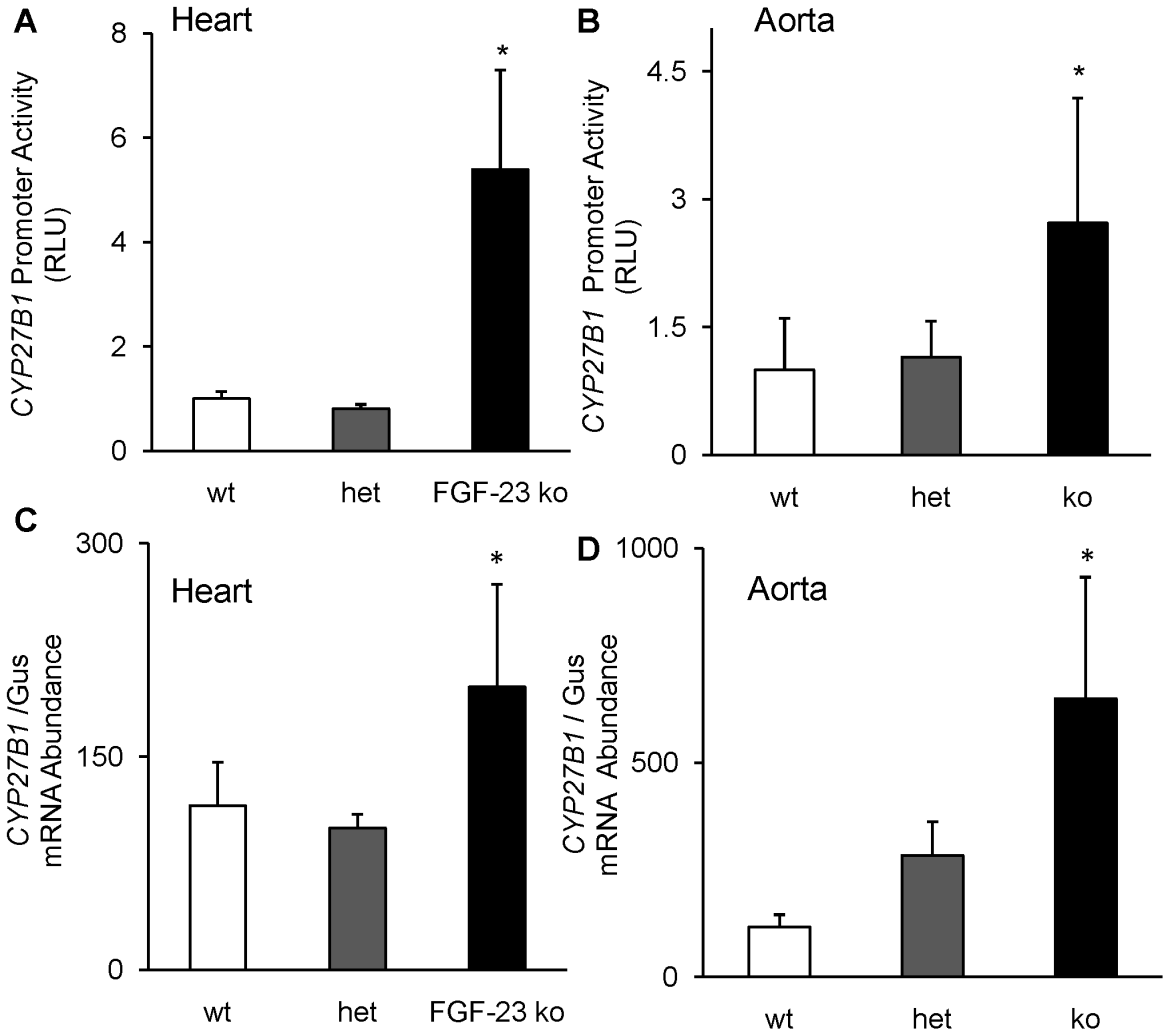
Cardiac and aortic expression of *CYP27B1*. *Fgf-23^+/+^/*1α-Luc*^+/−^* (wt), *fgf-23^+/−/^*1α-Luc*^+/−^* (het) and *fgf-23^−/−/^*1α-Luc*^+/−^* (ko) mice were bred as described in *Methods.* Mice were sacrificed at 4 weeks of age, the heart and aorta removed and divided into two parts for determination of *CYP27B1* promoter activity in heart **(A),** and aorta **(B)**, expressed as luciferase activity per mg of tissue. Graph depicts fold change with respect to luciferase activity in *fgf-23^+/+^/*1α-Luc*^+/−^* mice. *CYP27B1* mRNA expression in heart **(C),** and aorta **(D)**, quantitated by real-time PCR, normalized to that of gus mRNA, and expressed as a percent relative to *fgf-23^+/+^/*1α-Luc*^+/−^* mice. Bars depict mean±SEM (n = 5 mice/group) *P<0.05, compared to *fgf-23^+/+^/*1α-Luc*^+/−^* mice.

Autocrine and paracrine effects of 1,25(OH)_2_D have been well described in several tissues [26]–[28]. *CYP27B1* mRNA expression is detected in the heart [54], although its role in normal cardiac function or disease states is unknown. FGF-23 directly induces left ventricular hypertrophy in normal mice and stimulates hypertrophy of isolated cardiac myocytes, suggesting that the heart is a target tissue for FGF-23 action [55]. To determine whether FGF-23 regulates *CYP27B1* in extra-renal tissues, we utilized the *fgf-23^−/−/^*1α-Luc^+*/−*^ transgenic mouse model. We determined 1α-hydroxylase promoter activity and mRNA expression in the heart of *fgf-23^+/+^/*1α-Luc^+*/−*^, fgf*-23^+/−/^*1α-Luc^+*/−*^ and *fgf-23^−/−/^*1α-Luc^+*/−*^ transgenic mice at 4 weeks of age. In *fgf-23^−/−/^*1α-Luc^+*/−*^ transgenic mice, *CYP27B1* promoter-driven luciferase activity in the heart was 5-fold higher (Fig. 6A), and *CYP27B1* mRNA abundance was 2-fold higher (Fig. 6C) than in *fgf-23^+/+^/*1α-Luc^+*/−*^ mice. To demonstrate that FGF-23 can suppress cardiac *CYP27B1* mRNA expression, *fgf-23^−/−/^*1α-Luc^+*/−*^ mice received a single injection of FGF-23 (150ng/g) or vehicle. Cardiac *CYP27B1* mRNA expression decreased by 30% in the heart in FGF-23-treated mice compared to vehicle treated mice (194.30±84.35 vs 136.0±38.84, *P<0.05*). We also determined *CYP27B1* promoter activity and gene expression in the aorta. We demonstrate a 3- and 7-fold increase in *CYP27B1* promoter activity and mRNA expression, respectively, in the aorta of *fgf-23^−/−/^*1α-Luc^+*/−*^ transgenic mice compared to *fgf-23^+/+^/*1α-Luc^+*/−*^ mice (Fig. 6B and D). These studies provide evidence in mice lacking circulating FGF-23 that *CYP27B1* gene expression is transcriptionally upregulated in both the heart and aorta. Of note, we detected no significant changes in either *CYP27B1* promoter activity, or mRNA abundance in the heart and aorta in heterozygous (*fgf-23^+/−/^*1α-Luc^+*/−*^) mice compared to the control group. To determine whether regulation of *CYP27B1* expression was specific to FGF-23 action in target tissues, we administered basic FGF to *fgf-23^+/+^/*1α-Luc^+*/−*^ mice and determined *CYP27B1* mRNA abundance in the kidney and heart. Basic FGF is another member of the FGF family of proteins that can activate MAPK signaling in several target tissues. We observed no significant change in *CYP27B1* mRNA abundance in the kidney (89±20 in basic FGF-treated group vs 49±12 in vehicle-treated group, n = 4 mice/group, P = 0.22) or in the heart (98±20 in FGF2-treated group vs 108±28 in vehicle-treated group, n = 4 mice/group, P = 0.77). There was an upward trend (but not statistically significant) for *CYP27B1* mRNA abundance in the kidney of mice treated with basic FGF which was opposite in direction to that observed in mice treated with FGF-23.

**Figure 7:**
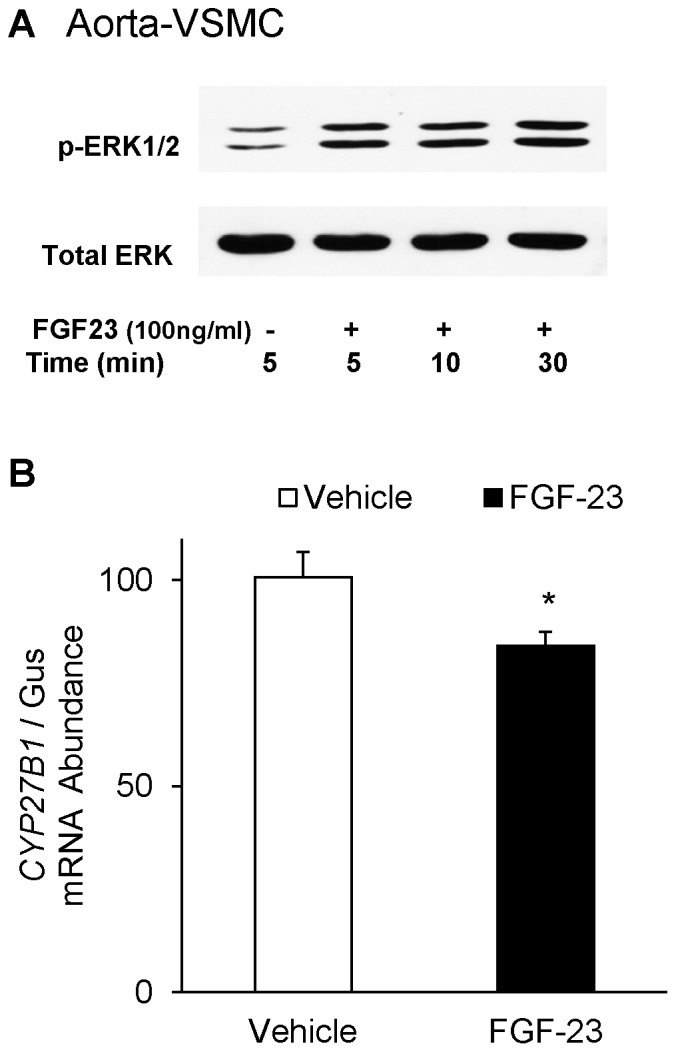
Effect of FGF-23 on *CYP27B1* mRNA expression in cultured mouse aortic vascular smooth muscle cells (VSMC). **A.** Activation of ERK1/2 signaling pathway was demonstrated in VSMC treated with FGF-23 (100 ng/ml) for 5–60 min. Phosphorylated ERK1/2 protein expression was detected by western blot analysis. Total Erk2 protein expression was used as loading control. **B.** *CYP27B1* mRNA expression in VSMC treated with FGF-23 (100 ng/ml) for 21 hrs. mRNA expression was quantitated by real-time PCR, normalized to that of gus mRNA, and expressed as a percent relative to vehicle-treated group. Bars depict mean±SEM (n = 3 separate experiments in triplicate) *P<0.05, compared to vehicle-treated group.

To demonstrate whether FGF-23 activates MAPK signaling via ERK1/2 and directly suppresses *CYP27B1* expression in the vascular system, we treated cultured mouse aortic vascular smooth muscle cells (VSMC) with FGF-23. We detected phosphorylation of ERK1/2 protein at 5–30 minutes in FGF-23-treated VSMC when compared to vehicle-treated cells (Fig. 7A). Furthermore, FGF-23 significantly suppressed *CYP27B1* mRNA abundance in cultured VSMC when compared to the vehicle-treated group (Fig. 7B). These studies provide evidence for a direct suppression of *CYP27B1* gene expression by FGF-23 in the aorta.

#### Other extra-renal sites

**Figure 8:**
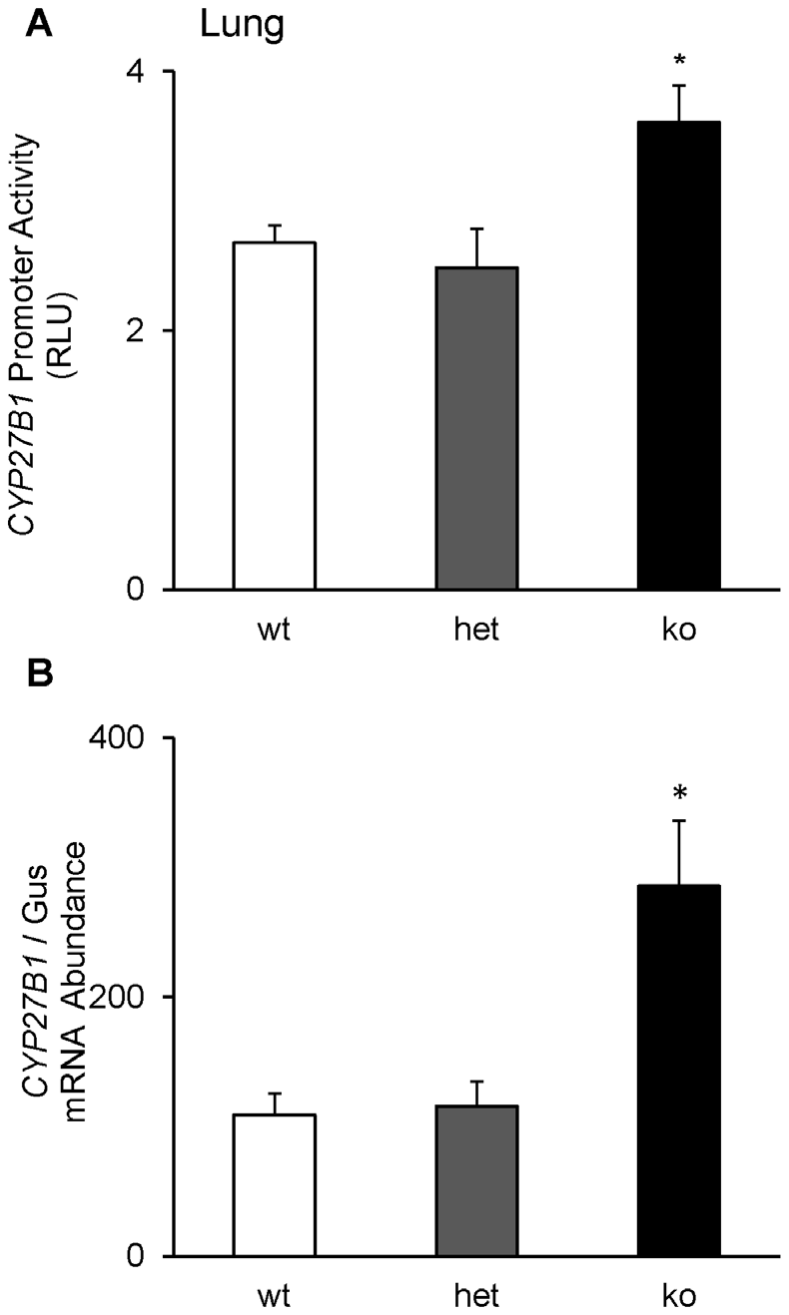
Pulmonary expression of *CYP27B1.* *Fgf-23^+/+^/*1α-Luc*^+/−^* (wt), *fgf-23^+/−/^*1α-Luc*^+/−^* (het) and *fgf-23^−/−/^*1α-Luc*^+/−^* (ko) mice were bred as described in *Methods.* Mice were sacrificed at 4 weeks of age and the lung removed and divided into two parts for determination of **A.** *CYP27B1* promoter activity, expressed as luciferase activity per mg of tissue. Graph depicts fold change with respect to luciferase activity in *fgf-23^+/+^/*1α-Luc*^+/−^* mice. **B.** *CYP27B1* mRNA expression, quantitated by real-time PCR, normalized to that of gus mRNA, and expressed as a percent relative to *fgf-23^+/+^/*1α-Luc*^+/−^* mice. Bars depict mean±SEM (n = 5 mice/group), *P<0.05, compared to *fgf-23^+/+^/*1α-Luc*^+/−^* mice.

**Figure 9:**
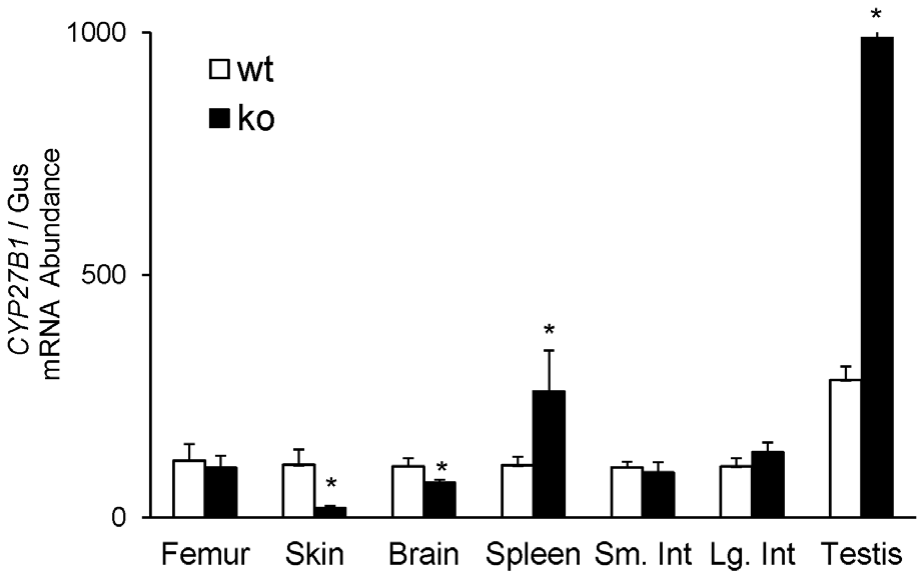
*CYP27B1* expression in extra-renal tissues of *fgf-23^+/+^/*1α-Luc*^+/−^* (wt) and *fgf-23^−/−/^*1α-Luc*^+/−^* (ko) mice. Mice were sacrificed at 4 weeks of age and the organs removed for determination of *CYP27B1* mRNA expression, quantitated by real-time PCR, normalized to that of gus mRNA, and expressed as a percent relative to *fgf-23^+/+^/*1α-Luc*^+/−^* mice. Bars depict mean±SEM (n = 5 mice/group), *P<0.05, compared to *fgf-23^+/+^/*1α-Luc*^+/−^* mice. Sm.Int- small intestine, Lg.Int-large intestine.

To identify novel target tissues in which *CYP27B1* expression might be up regulated in the absence of circulating FGF-23, we determined *CYP27B1* mRNA expression in *fgf-23^−/−/^*1α-Luc^+*/−*^ mice in several extra-renal tissues. In the lung, *CYP27B1* promoter-driven luciferase activity was 35% higher (Fig. 8A) and *CYP27B1* mRNA was 3-fold higher (Fig. 8B) in *fgf-23^−/−/^*1α-Luc mice compared to *fgf-23^+/+^/*1α-Luc^+*/−*^ mice. In the spleen, *CYP27B1* mRNA was 3-fold higher and in the testis was 10-fold higher (Fig. 9) in *fgf-23^−/−/^*1α-Luc^+*/−*^ mice than in control mice; there were no significant difference in *CYP27B1* mRNA in these tissues in the heterozygous mice (*fgf-23^+/−/^*1α-Luc^+*/−*^) when compared to the control group (data not shown). In contrast to the increased *CYP27B1* expression in lung, spleen and testis of *fgf-23^−/−/^*1α-Luc^+*/−*^ mice, *CYP27B1* mRNA in skin and brain in these animals were lower by 80% and 28%, respectively, compared to control mice (Fig. 9). There was no significant change in *CYP27B1* mRNA expression in the small and large intestine and femur (Fig. 9), extra-renal tissues reported to synthesize 1,25(OH)_2_D [47]. In the kidney, expression of *CYP24A1* mRNA was 63% lower in *fgf-23^−/−/^*1α-Luc^+*/−*^ mice compared to control mice (110.46±23.84 vs 40.43±6.44, *P*<0.05). *CYP24A1* mRNA expression increased by 13-fold (1574.42±539.59 vs118.98±45.93, *P*<0.05) in the small intestine, by 21-fold (2441.99±603.56 vs 113.30±33.01, *P*<0.05) in the large intestine, and by 3-fold (655.80±141.46 vs 222.99±36.13, *P*<0.05) in the testis in *fgf-23^−/−/^*1α-Luc^+*/−*^ mice compared to the control group. No significant differences in *CYP24A1* mRNA were observed in the other tissues examined.

### Serum Biochemical Values in *fgf-23^−/−/^1α-Luc^+/−^* Mice

Hypocalcemia, hypophosphatemia and increased PTH concentrations are known inducers of renal *CYP27B1* gene expression in mice. To determine whether changes in serum concentrations of Ca, Pi, or PTH might explain the difference in *CYP27B1* gene expression between *fgf-23^−/−/^1α-Luc^+/−^* and *fgf-23^+/+^/*1α-Luc^+*/−*^ mice, we determined their serum concentrations in these animals. In *fgf-23^−/−/^1α-Luc^+/−^* mice, the mean concentrations of serum Ca and Pi were significantly higher when compared to values in control mice (Table 1). PTH concentrations did not differ between the groups of mice. Moreover, administration of FGF-23 to *fgf-23^−/−/^1α-Luc^+/−^* mice did not significantly alter serum Ca, Pi or PTH concentrations suggesting that the changes in *CYP27B1* gene expression in these mice are due to the direct action of FGF-23 in target tissues and not due to changes in serum Ca, Pi or PTH.

**Table 1:** Serum biochemical values in *fgf-23^+/+^/*1α-Luc*^+/−^* (wt) and *fgf-23^−/−/^*1α-Luc*^+/−^* (ko) transgenic mice.

Mouse	Serum Ca (mg/dl)	Serum Pi (mg/dl)	Serum PTH (pg/ml)
*Fgf-23^+/+^/*1α-Luc*^+/−^* (wt)	9.0±0.3	9.5±0.5	40.1±7.8
*Fgf-23^−/−/^*1α-Luc*^+/−^* (ko) (vehicle-treated)	10.9±0.3*	16.0±0.4*	29.9±3.8
*Fgf-23^−/−/^*1α-Luc*^+/−^* (ko) (FGF-23-treated)	11.1±0.7*	17.1±0.9*	31.2±5.2

* *P<0.05 when compared to fgf-23^+/+^/*1α-Luc*^+/−^* (wt) mice. Values expressed as Mean±SEM, n = 7 mice per group.

## Discussion

We provide evidence that *CYP27B1* is transcriptionally regulated by FGF-23 in the kidney, using both an *in vitro* and *in vivo* promoter-driven luciferase reporter system. We demonstrate that MAPK signaling via MEK/ERK1/2 plays a critical role in the transcriptional regulation of *CYP27B1* in the kidney. In addition, we identify novel tissue targets for FGF-23-dependent regulation of *CYP27B1* expression, specifically, the heart, aorta, spleen, lung, skin, brain and testis.

After the human *CYP27B1* gene was cloned [10], [11], promoter activity of the 5′ flanking region (∼1.5 kb length) was examined *in vitro* in modified pig kidney (AOK-B50) [38],[40] mouse renal proximal tubule (MCT, MPCT) [32], [39], [56] and HEK-293 [57], [58] cells. Here, we studied transcriptional activity of the human *CYP27B1* gene in HEK-293 cells transfected with 1.6 kb of 5′ flanking DNA. We chose HEK-293 cells because they are more easily transfected than other cell lines, and signaling by FGF-23 has been well described in this system [41], [59]. In HEK-293 cells, basal activity of the 1.6 kb human *CYP27B1* promoter was 170-fold higher than that of the promoterless vector, and the activity remained high with −1.1 kb, −926 bp and −409 bp deletion constructs. We observed a 3-fold reduction in basal activity of the −789 bp and −200 bp deletion constructs when compared to the full-length 1.6 kb promoter, suggesting the presence of enhancers in the regions from −926 bp to −789 bp and −409 bp to −200 bp. Our findings are similar to those described previously in HEK-293 cells [57], although the *CYP27B1* promoter deletion constructs differed by a few base pairs in the two studies. We demonstrate that FGF-23 induced a dose-dependent suppression of *CYP27B1* promoter activity with a maximum suppression of 70%. The suppression of *CYP27B1* promoter activity by FGF-23 was seen in all the deletion constructs examined, suggesting that the regulatory region for FGF-23 lies within the first 200 bp of 5′ flanking DNA. Thus, regulation of renal *CYP27B1* expression by FGF-23 occurs at least in part by transcriptional mechanisms, similar to the regulation induced by PTH, calcitonin and 1,25(OH)_2_D in the kidney [38]–[40], [56], [58].

Having demonstrated transcriptional regulation of *CYP27B1* by FGF-23 *in vitro*, we sought to demonstrate regulation *in vivo* by utilizing 1α-Luc transgenic mice, in which dietary restriction of calcium and vitamin D [45]–[47] induces appropriate increases in renal *CYP27B1* transgene expression. We found that in the *fgf-23^−/−/^*1α-Luc^+*/−*^ mice, renal *CYP27B1* promoter activity in the kidney was increased by 250% compared with that in mice bearing an intact fgf-23 gene. The induction of *CYP27B1* promoter activity was accompanied by concomitant increases in renal expression of *CYP27B1* mRNA and protein. Of note, the increased *CYP27B1* expression in fgf-23 null mice cannot be explained by their increased serum concentrations of phosphorus, calcium or 1,25(OH)_2_D (which are characteristic features of fgf-23 null mice), as these factors suppress *CYP27B1* expression. With administration of FGF-23, renal *CYP27B1* promoter activity and protein abundance decreased significantly, and the suppressive effect was blocked by pre-treatment with a MEK inhibitor, PD0325901. We also observed that FGF-23-induced suppression of *CYP27B1* promoter activity was blocked by MEK inhibition in HEK 293 cells transfected with the 200 bp deletion construct. Thus, the present findings provide evidence that regulation of renal *CYP27B1* expression by FGF-23 is mediated at least in part by transcriptional mechanisms via activation of the ERK1/2 signaling pathway, both *in vivo* and *in vitro*.

Circulating 1,25(OH)_2_D has effects on cardiovascular tissue: 1,25(OH)_2_D can decrease myocardial proliferation and inhibit ventricular hypertrophy in cultured cells and in experimental animals [60]–[63]. In patients with chronic kidney disease, treatment with 1,25(OH)_2_D is associated with reduced risk of cardiovascular death [64], [65]. *CYP27B1* expression is detected in the heart [54], [66] but whether local 1,25(OH)_2_D synthesis contributes to normal functioning of the myocardium is not known. 1α-hydroxylase enzyme activity is present in vascular smooth muscle cells and is regulated by PTH [67]; however, whether local 1,25(OH)_2_D synthesis contributes to the normal function of vascular smooth muscle cells is unknown. Recently, the myocardium [55] and vascular smooth muscle cells [68] were identified as targets for FGF-23 action. In the heart, FGF-23 induces left ventricular hypertrophy independent of its co-factor, klotho, and in vascular smooth muscle cells, FGF23 inhibits vascular calcification, an effect that is klotho-dependent. In the present study, we provide evidence that FGF-23 down-regulates *CYP27B1* in the heart and aorta in fgf-23 null mice. In *fgf-23^−/−/^*1α-Luc*^+/−^* transgenic mice, we demonstrate in the heart a 500% increase in *CYP27B1* promoter activity and 200% increase in mRNA expression, and in the aorta, we demonstrate a 300% increase in *CYP27B1* promoter activity and a 700% increase in mRNA expression when compared to those values in tissues from control mice. Furthermore, we demonstrate in cultured mouse aortic vascular smooth muscle cells that FGF-23 activates ERK1/2 signaling and directly suppresses *CYP27B1* mRNA expression. We thus demonstrate for the first time, that FGF-23 suppresses *CYP27B1* gene transcription in cardiovascular tissue. Whether regulation of *CYP27B1* gene expression by FGF-23 in cardiovascular tissue plays a role in the myocardial or vascular structure or function requires further investigation.

The parathyroid gland expresses klotho and is a target for FGF-23 action which is to inhibit PTH secretion and stimulate *CYP27B1* expression [29], [69], [70]. The stimulation of *CYP27B1* expression by FGF-23 in the parathyroid gland is in sharp contrast with its suppressive effect on *CYP27B1* expression in the kidney. Similar to its effect in the parathyroid gland, we found that in the brain and skin of *fgf-23^−/−^* mice, absence of circulating FGF-23 down-regulated *CYP27B1* mRNA expression which is opposite to the direction of change in *CYP27B1* mRNA expression in the kidney, heart, aorta, lung, spleen and testis in these mice. Since 1α-hydroxylase enzyme activity in extra-renal tissues is an important source of 1,25(OH)_2_D for its autocrine and paracrine functions [26]–[30], further investigation is necessary to determine whether FGF-23-induced regulation of *CYP27B1* in extra-renal sites plays a role in the normal function of these tissues. In the lung, increased *CYP27B1* mRNA expression in alveolar macrophages of patients with sarcoidosis [22], [71], [72] contributes to increased pulmonary production of 1,25(OH)_2_D. However, whether pulmonary 1,25(OH)_2_D production plays a role in regulating normal biological functions in the lung is unknown. Similarly, the *CYP27B1* gene is expressed in the testis [73] but whether testicular 1,25(OH)_2_D production plays a role in regulating biological functions in the testis is unknown. Here, for the first time we show a significant up-regulation of *CYP27B1* expression in the lung and testis in mice lacking circulating fgf-23. It is of interest that fgf-23 null mice demonstrate pathological changes in the lung and testes that are rescued by global deletion of the *CYP27B1* in the double mutant *fgf-23^−/−/^1α- hydroxylase^−/−^* mouse [49]. Specifically, *fgf-23^−/−^* mice develop pulmonary emphysema and significant testicular atrophy and infertility that are notably ameliorated in the double mutant *fgf-23^−/−/^1α- hydroxylase^−/−^* mouse [49]. Whether the improvement in pathological changes in *fgf-23^−/−/^1α- hydroxylase^−/−^* mice are induced by significant reductions in circulating serum 1,25(OH)_2_D concentrations or decreased local synthesis of 1,25(OH)_2_D in the lung and testis could not be determined in the above mentioned study. Thus, the contribution of local 1,25(OH)_2_D synthesis to the biological functions of the lung and testis remain to be determined.

In our study, absence of FGF-23 did not affect expression of *CYP27B1* mRNA in the intestine and bone, which are important extra-renal sites for local 1,25(OH)_2_D synthesis. However, absence of FGF-23 was associated with a dramatic increase in intestinal expression of *CYP24A1* mRNA. Such up-regulation of *CYP24A1* mRNA expression might be mediated by the high circulating 1,25(OH)_2_D concentrations and could mitigate 1,25(OH)_2_D-mediated intestinal absorption of calcium and phosphorus. In summary, we demonstrate that regulation of *CYP27B1* by FGF-23 occurs at least in part via transcriptional mechanisms in kidney and extra-renal tissues and that ERK1/2 signaling plays an important role in mediating FGF-23 action. We have identified the heart, aorta, and several other sites as novel target tissues for FGF-23-induced regulation of *CYP27B1*. Further studies are necessary to understand the role of FGF-23-mediated regulation of *CYP27B1* in these extra-renal sites.
